# Endoglin Is an Endothelial Housekeeper against Inflammation: Insight in ECFC-Related Permeability through LIMK/Cofilin Pathway

**DOI:** 10.3390/ijms22168837

**Published:** 2021-08-17

**Authors:** Elisa Rossi, Alexandre Kauskot, François Saller, Elisa Frezza, Sonia Poirault-Chassac, Anna Lokajczyk, Pierre Bourdoncle, Bruno Saubaméa, Pascale Gaussem, Miguel Pericacho, Regis Bobe, Christilla Bachelot-Loza, Samuela Pasquali, Carmelo Bernabeu, David M. Smadja

**Affiliations:** 1 Faculty of Pharmacy, University of Paris, F-75006 Paris, France; elisa.frezza@parisdescartes.fr (E.F.); sonia.poirault-chassac@inserm.fr (S.P.-C.); anna.lokajczyk@parisdescartes.fr (A.L.); bruno.saubamea@parisdescartes.fr (B.S.); pascale.gaussem@aphp.fr (P.G.); christilla.bachelot-loza@parisdescartes.fr (C.B.-L.); samuela.pasquali@parisdescartes.fr (S.P.); david.smadja@aphp.fr (D.M.S.); 2 IThEM, Inserm UMR-S 1140, F-75006 Paris, France; 3 HITh, UMR-S 1176, INSERM—Faculty of Medicine, University Paris-Saclay, F-94270 Le Kremlin-Bicêtre, France; alexandre.kauskot@inserm.fr (A.K.); francois.saller@universite-paris-saclay.fr (F.S.); regis.bobe@inserm.fr (R.B.); 4 CiTCoM, CNRS, Université de Paris, F-75006 Paris, France; 5 Plate-Forme IMAG’IC Institut Cochin Inserm U1016-CNRS UMR8104, Université Paris Descartes, F-75006 Paris, France; pierre.bourdoncle@inserm.fr; 6 UMR-S 1144, F-75006 Paris, France; 7 AP-HP, Hematology Department, Hôpital Européen Georges Pompidou, F-75015 Paris, France; 8 Department of Physiology and Pharmacology, Universidad de Salamanca, 37008 Salamanca, Spain; pericacho@usal.es; 9 Centro de Investigaciones Biológicas Margarita Salas, 28040 Madrid, Spain; bernabeu.c@cib.csic.es; 10 Consejo Superior de Investigaciones Científicas (CSIC) and Centro de Investigación Biomédica en Red de Enfermedades Raras (CIBERER), 28040 Madrid, Spain; 11 Biosurgical Research Lab (Carpentier Foundation), F-75000 Paris, France

**Keywords:** endoglin, ECFC, cofilin, HHT1, TNFα

## Abstract

Endoglin (Eng) is an endothelial cell (EC) transmembrane glycoprotein involved in adhesion and angiogenesis. Eng mutations result in vessel abnormalities as observed in hereditary hemorrhagic telangiectasia of type 1. The role of Eng was investigated in endothelial functions and permeability under inflammatory conditions, focusing on the actin dynamic signaling pathway. Endothelial Colony-Forming Cells (ECFC) from human cord blood and mouse lung/aortic EC (MLEC, MAEC) from *Eng^+/+^* and *Eng^+/−^* mice were used. ECFC silenced for Eng with Eng-siRNA and ctr-siRNA were used to test tubulogenesis and permeability +/− TNFα and +/− LIM kinase inhibitors (LIMKi). In silico modeling of TNFα–Eng interactions was carried out from PDB IDs 5HZW and 5HZV. Calcium ions (Ca^2+^) flux was studied by Oregon Green 488 in epifluorescence microscopy. Levels of cofilin phosphorylation and tubulin post-translational modifications were evaluated by Western blot. F-actin and actin–tubulin distribution/co-localization were evaluated in cells by confocal microscopy. Eng silencing in ECFCs resulted in a decrease of cell sprouting by 50 ± 15% (*p* < 0.05) and an increase in pseudo-tube width (41 ± 4.5%; *p* < 0.001) compared to control. Upon TNFα stimulation, ECFC Eng–siRNA displayed a significant higher permeability compared to ctr-siRNA (*p* < 0.01), which is associated to a higher Ca^2+^ mobilization (*p* < 0.01). Computational analysis suggested that Eng mitigated TNFα activity. F-actin polymerization was significantly increased in ECFC Eng-siRNA, MAEC^+/−^, and MLEC^+/−^ compared to controls (*p* < 0.001, *p* < 0.01, and *p* < 0.01, respectively) as well as actin/tubulin distribution (*p* < 0.01). Furthermore, the inactive form of cofilin (P-cofilin at Ser3) was significantly decreased by 36.7 ± 4.8% in ECFC Eng-siRNA compared to ctr-siRNA (*p* < 0.001). Interestingly, LIMKi reproduced the absence of Eng on TNFα-induced ECFC-increased permeability. Our data suggest that Eng plays a critical role in the homeostasis regulation of endothelial cells under inflammatory conditions (TNFα), and loss of Eng influences ECFC-related permeability through the LIMK/cofilin/actin rearrangement-signaling pathway.

## 1. Introduction

Endoglin (Eng; 180-kDa), alias CD105, is a transmembrane glycoprotein expressed on endothelial cells (EC), acting as an auxiliary receptor for transforming growth factor β (TGF-β), binding bone morphogenetic protein 9 (BMP9) and BMP10 [1,2,3,4], and playing a key role in vascular physiology, angiogenesis, and vascular remodeling [5,6]. Heterozygous mutations in the Eng gene lead to a lower expression of the protein on the EC surface. They are responsible for Hereditary Hemorrhagic Telangiectasia (HHT) type I, which is a vascular pathology characterized by epistaxis, mucocutaneous, or gastrointestinal telangiectasia, and pulmonary, cerebral, or hepatic arteriovenous malformations (AVM) [7,8]. In addition to its recognized role as a TGF-β co-receptor, Eng may also impact EC behavior by regulating cell adhesion via a non-canonical TGF-β pathway [9] and by interacting with leukocytes, platelets, or vascular mural cell integrins through its pro-adhesive Arg-Gly-Asp motif (RGD) [10,11,12]. Furthermore, recent mouse pre-clinical data suggested that membrane Eng plays a role in the regulation of EC permeabilization [11] and that Eng deficiency leads to EC hyper-permeability through the constitutive activation of RhoA, a destabilization of endothelial barrier function, and a reduction of VE-cadherin [13]. Interestingly, RhoA is expressed in ECs and is involved in the pathological angiogenesis of retinal diseases [14]. In addition, dysregulated levels of membrane-bound endoglina or soluble endoglin in the retina modulate vascular remodeling, the formation of AVMs, and vascular permeability [11,15,16,17]. Furthermore, elevated levels of soluble endoglin have been reported in the aqueous humor of different retinopathies [18,19], endoglin being a promising therapeutic target in these pathological conditions [20]. 

Eng is expressed on endothelial colony-forming cells (ECFCs) [21]. These cells are progenitors displaying robust vasculogenic properties [22]. Intriguingly, Eng-decreased expression in HHT1 seems to compromise the ECFC cytoskeleton organization [23], but the exact mechanism involved remains unknown. Among the proteins involved in actin redistribution, cofilin-1, a non-muscle type of cofilin [24], is an actin-binding protein that is able to disassemble actin filaments and to bind actin monomers and filaments by targeting G- and F-actin, respectively [25]. Since the role of Eng in ECFC vasculogenic properties and the potential link with the cytoskeleton are still incompletely understood, we investigated how the loss of Eng might compromise ECFC cytoskeleton organization and permeability in basal and pro-inflammatory conditions and its possible functional relationships with cofilin.

## 2. Results

### 2.1. Endoglin Regulates ECFC Tubulogenesis and Permeability under Inflammatory Conditions

**Figure 1:**
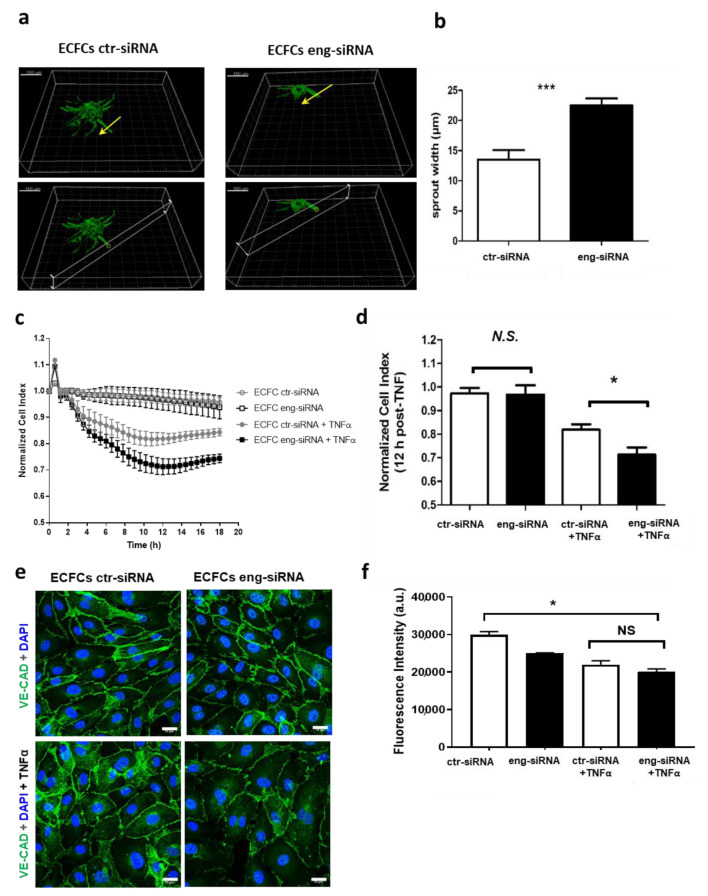
Endoglin silencing affects vessel sprout diameter and permeability. (**a**) Three-dimensional (3D) reconstruction of ECFC ctr-siRNA and ECFC Eng-siRNA to evaluate sprout diameter. In green, Actin F (Alexa 488), and in red, nuclei (TOPRO-3) are stained (scale bar, 150 µm). (**b**) When Eng is suppressed, sprouts are wider than in controls (*** *p* < 0.001). (**c**) Using a real-time impedance-based cell analyzer (iCELLigence system, ACEA Biosciences, San Diego, USA), ECFC ctr-siRNA and ECFC Eng-siRNA are analyzed in basal condition and under 20 ng/mL TNFα stimulation (gray and black lines, respectively). As shown by the quantification in (**d**), a significant difference between ECFC ctr-siRNA (white column) and ECFC Eng-siRNA (black column) is found 12 h after TNFα stimulation (* *p* < 0.05). (**e**) Immunofluorescence staining for VE-CAD in ECFC-ctr and ECFC Eng-siRNA with the absence of or in the presence of 20 ng/mL TNFα for 1 h (scale bar, 22 µm). (**f**) Quantification of the pictures in (**c**) does not show differences between control and Eng-siRNA in terms of VE-CAD membrane staining nor of fluorescence intensity in the presence of TNFα (* *p* < 0.05).

Tubulogenesis assay used ECFCs transfected with Eng-siRNA or ctr-siRNA. Silencing was considered effective during the time course of the experiment since the expression of Eng protein decreased below 20% at D1 and remained around 50% at D4 (Figure S1a,b). Eng silencing significantly decreased tubulogenesis with a reduction of sprouting by 54 ± 7% (* *p* < 0.05), as observed by confocal microscopy of beads covered by ECFC Eng-siRNA. Pseudo-tube numbers per bead were also significantly decreased (42% ± 2.2 *p* < 0.05) (Videos S1 and S2 in Supplementary Materials; Figure S1c,d), whereas their width was significantly increased by 41 ± 4.5% (*p* < 0.001) compared to ctr-siRNA ECFC (Figure 1a,b; Videos S1 and S2 in Supplementary Materials). As a whole, our data demonstrate that Eng silencing decreases tubulogenesis and increases sprout diameter.

Eng-silenced ECFC permeability was evaluated in basal conditions and in the presence of TNFα. In the absence of TNFα treatment, Eng silencing had no effect on cell permeability (Figure 1c,d). Inflammatory conditions induced by TNFα treatment increased ECFC permeability (Figure 1c,d) with a significant difference between Eng-siRNA (0.7 ± 0.02, *p* < 0.05), compared to ctr-siRNA (0.85 ± 0.02) (*p* < 0.05), suggesting that the absence of Eng favors cell permeability in inflammatory conditions. However, this finding was independent from VE-CAD expression that did not differ between ECFC Eng-siRNA and ctr-siRNA (Figure 1e,f), which is in line with the findings of Jerkic and Letarte in mouse EC derived from *Eng^+/−^* and *Eng^+/+^* [13]. 

Since an increase in cytosolic Ca^2+^ was shown to regulate endothelial permeability [26], the role of Eng silencing in this process was investigated using the Ca^2+^-sensitive dye Oregon Green 488 BAPTA 1-AM. Under 20 ng/mL TNFα stimulation, in the presence of extracellular Ca^2+^, a higher level of intracellular Ca^2+^ was observed in ECFC Eng-siRNA compared to ctr-siRNA (*p* < 0.05). The same results were obtained in the absence of added Ca^2+^, in favor of a role of Eng in Ca^2+^ mobilization (*p* < 0.01) (Figure S2a,b; Videos S3 and S4 in Supplementary Materials).

### 2.2. In Silico Interaction between Endoglin and TNFα

**Figure 2:**
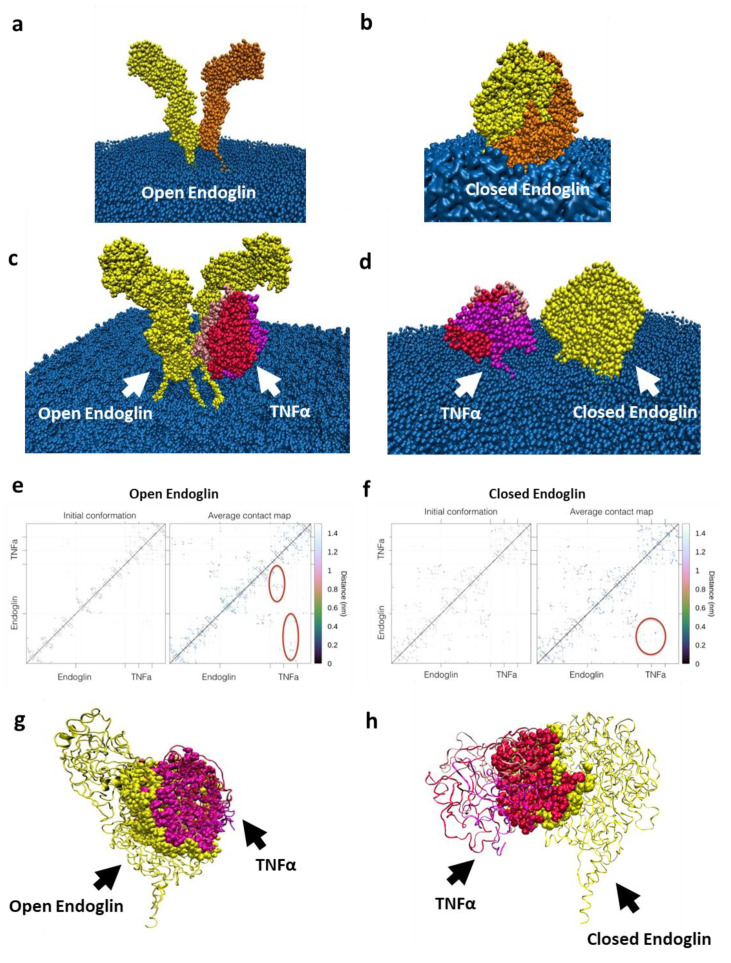
TNFα and endoglin interaction. (**a**) Endoglin alone embedded in the membrane. The two arms of the molecule are colored in yellow and orange, respectively, to show the two homodimeric parts of the molecule. (**b**) Endoglin during the simulation in its closed form with the two arms wrapping around each other. (**c**) Initial configuration of TNFα ligand (trimeric form in red and pink) and endoglin (homodimeric form in yellow) in “open” conformation. Endoglin is embedded in the phospholipidic membrane (blue). (**d**) Initial conformation of TNFα ligand and endoglin in “closed” conformation. (**e**) Contact maps of the TNFα and open endoglin in the initial conformation (right) and of the average computed over the whole simulation (left). The circles in red highlight the region of contact of the aggregate. (**f**) Contact maps of the TNFα and closed endoglin in the initial conformation (right) and of the average computed over the whole simulation (left). The circle in red highlights the region of contact of the aggregate. (**g**) Representative surface contact between TNFα and open endoglin; 137 residues of TNFα are in contact with endoglin. (**h**) Representative surface contact between TNFα and closed endoglin; 54 residues of TNFα are in contact with endoglin, while 21 residues are in contact in both the “open” and “closed” simulations.

No direct interaction between TNFα and membrane-bound Eng has been described so far; thus, we postulated the existence of such a physical interaction that could explain the increased permeability induced by TNFα, especially when Eng is inhibited (Figure 1c,d). This hypothesis was tested with an in silico approach. Computational analysis of membrane Eng showed that in the absence of any ligand, the molecule adopted two possible conformations, switching constantly from a so-called “open” to a “closed” form and vice versa (Figure 2a,b). The next step was to consider membrane Eng in the presence of TNFα (Figure 2c–f). Simulations with TNFα used Eng in both open and closed forms (Figure 2c,d). Contact maps of the TNFα and open Eng in the initial conformation and of the average computed over the whole simulation are shown in Figure 2e left and right, respectively. The circles in red highlight the region of contact of the aggregate observed during the simulation (Figure 2e; Video S5 in Supplementary Materials). The surface contact between Eng and TNFα involved 137 TNFα residues (Figure 2g; Video S6 in Supplementary Materials). Contact maps of the TNFα and closed Eng in the initial conformation and of the average computed over the whole simulation are shown in Figure 2f. In the closed simulation, the two macromolecules rapidly came into contact (Figure 2d,f,h and Videos S7 and S8 in Supplementary Materials) and formed a permanent aggregate. Our simulations showed that the most probable conformation of the molecule alone, in absence of ligand, is the closed form and that the contact between its monomers is constant (Figure S3a and Video S9 in Supplementary Materials). The same analysis was performed for the TNFα ligand alone, showing that the structure formed by the three chains is maintained thought a long simulation (Figure S3b and Video S10 in Supplementary Materials). Of note, in the open simulation, TNFα formed a very stable aggregate with the two arms of Eng accommodating for it with an extensive contact area, as is visible in Figure S4a–d. Late configurations showed that TNFα inserted deeply into the Eng cavity (Figure S4(b1,b2),c,d). Considering the closed form of Eng in the early simulation (Figure S4f), the surface area involved 28 TNFα residues in contact with Eng (Figure S4h), while later in the simulation, 54 TNFα residues were in contact with Eng (Figure S4g,i). It is to be noticed that 21 shared residues are in contact in both the open and closed simulations. These results suggest that TNFα can form stable aggregates with Eng and that the complex formed by soluble TNFα and Eng in its open form is particularly stable as TNFα finds a large complementary binding surface.

### 2.3. Endoglin Regulates Actin Dynamics 

**Figure 3:**
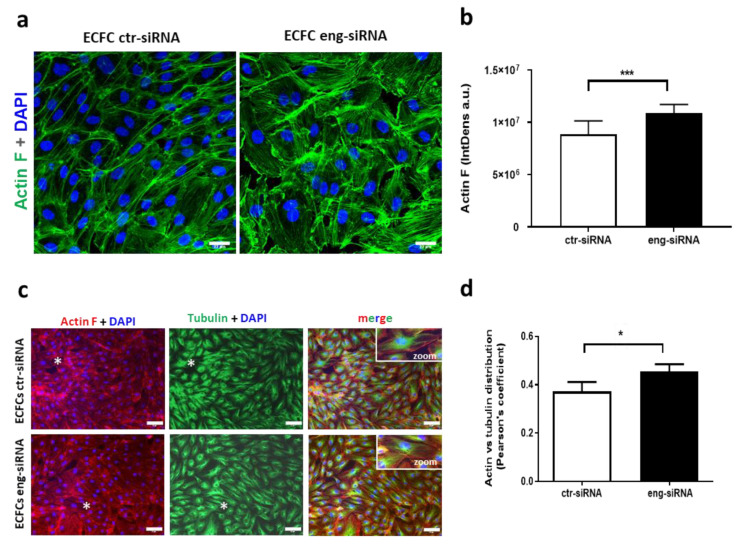
Endoglin silencing affects F-actin polymerization. (**a**) Immunofluorescence of F-actin (green, Phalloidin-Alexa 488) in ECFC ctr-siRNA and Eng-siRNA (scale bar, 22 µm). (**b**) Quantification of (**a**) (*** *p* < 0.001). (**c**) Co-staining of F-actin (red, Phalloidin-Alexa 546) and tubulin (green) (scale bar, 33 µm). (**d**) Evaluation of F-actin vs. tubulin distribution, using Pearson’s coefficient (* *p* < 0.05).

Actin cytoskeleton rearrangement plays an important role in maintaining endothelial barrier integrity [27]. Thus, prompted by the reported involvement of Eng in the actin cytoskeleton [23,28,29], we assessed its specific role in ECFC cytoskeleton. Eng silencing induced a significant increase in F-actin stress fiber formation compared to ECFC ctr-siRNA (*** *p* < 0.001) (Figure 3a,b). In addition, the organization of actin and tubulin filaments revealed a statistically significant difference of their co-localization in Eng-siRNA compared to control cells (* *p* < 0.05, Pearson’s coefficient) (Figure 3c,d). 

**Figure 4:**
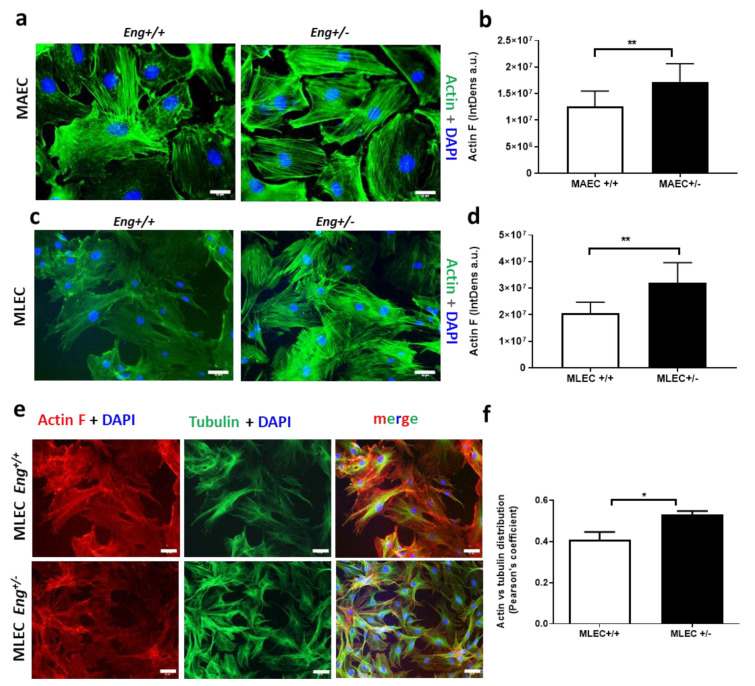
Murine endoglin in MAECs and MLEC is involved in F-actin polymerization and distribution. (**a**,**b**) Mouse aortic endothelial cells (MAEC) from a model of HHT1, stained for F-actin (green, Alexa 488) and DAPI (**a**), and its quantification (**b**). (**c**,**d**) Mouse lung endothelial cells (MLEC) from a model of HHT1 stained for F-actin (green, Alexa 488) and DAPI (**c**), and its quantification (**d**). A significant difference (** *p* < 0.01) between control and *Eng^+/−^* conditions was found in terms of actin distribution (**b**,**d**) by Image J considering fluorescence (IntDens). (**e**,**f**) *Eng^+/−^* MLEC stained for actin and tubulin (**e**) and their co-localization evaluated by Person’s coefficient (* *p* < 0.05) (**f**). (scale bar, 33 µm).

Similar results were obtained with MAEC and MLEC obtained from an Eng-deficient mouse model of HHT1, both in terms of actin polymerization (*p* < 0.01) (Figure 4a–d) and distribution (*p* < 0.05, Pearson’s coefficient) (Figure 4e,f), confirming an important role for Eng in actin dynamics. 

**Figure 5:**
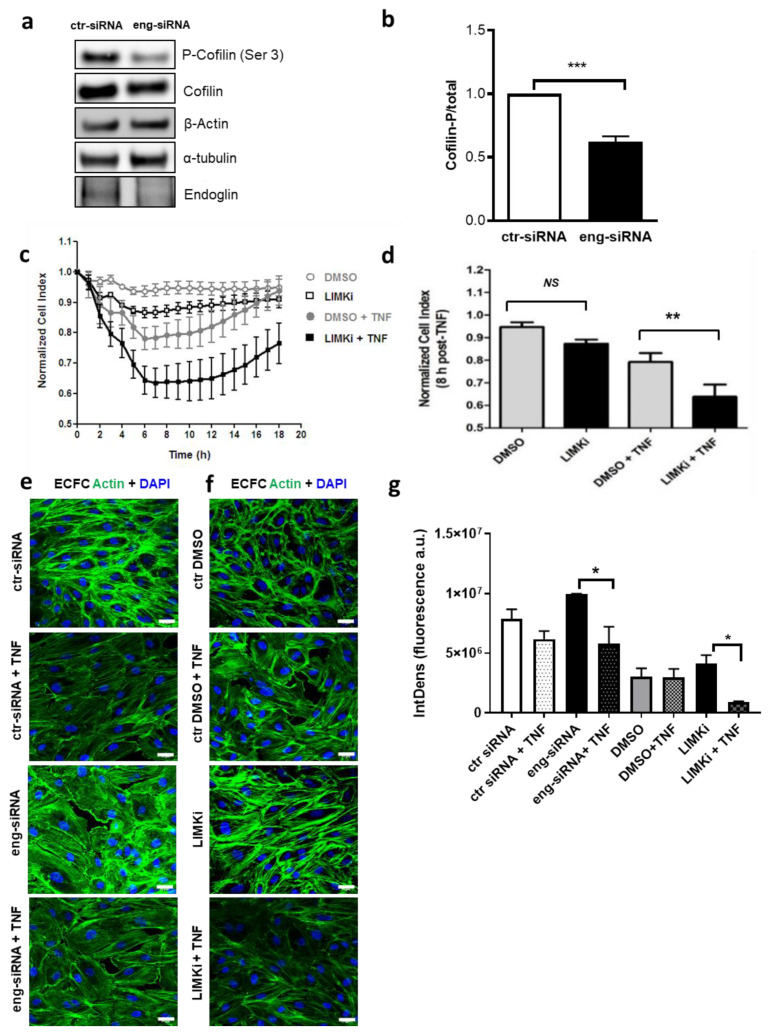
Endoglin silencing affects cofilin dynamics. (**a**,**b**) Western blot performed on *n* = 5 different clones of ECFC at passage 3–4, <30 days. A representative experiment is shown. Eng silencing in ECFC reduces P-Cofilin, suggesting an increased activity of cofilin when Eng is downregulated. Actin and tubulin were used as loading controls. The last row displays the reduced expression of Eng after siRNA silencing. (**b**) Quantification of five different ECFC clones shows a significant difference (*** *p* < 0.001) between ctr-siRNA and Eng-siRNA in the P-Cofilin/Total-Cofilin ratio. (**c**). Using a real-time impedance-based cell analyzer (iCELLigence system, ACEA Biosciences), ECFC controls (DMSO) and ECFC treated by LIMKi were analyzed in basal condition and under 20 ng/mL TNFα stimulation (gray and black lines, respectively). As shown by the quantification in (**d**), a significant difference between control ECFC (gray bar) and ECFC treated with LIMKi (black bar) 12 h after TNFα stimulation (** *p* < 0.01). (**e**,**f**) Immunofluorescence for F-actin in ECFC controls vs. ECFC Eng-siRNA +/−TNFα (**e**) and ECFC controls vs. ECFC LIMKi +/−TNFα (**f**). (scale bar, 22 µm). (**g**) The quantification of (**e**) and (**f**) confirms that ECFC Eng-siRNA and ECFC LIMKi display the same modifications in terms of F-actin (* *p* < 0.05).

Since cofilin has been described to interact with actin, therefore, we analyzed the role of Eng on the cofilin expression/activity. Interestingly, Eng silencing in ECFC decreased the level of the inactive form of cofilin (phosphorylated at Ser3) by 36.7 ± 4.8% (*p* < 0.001) (Figure 5a,b). Since LIMK is the kinase that phosphorylates cofilin, we investigated whether the LIMK/cofilin signaling pathway contributed to ECFC permeability by using a LIMK inhibitor (LIMKi) [18]. Interestingly, in the absence of TNFα, the inhibition of cofilin phosphorylation by LIMKi had no effect on cell permeability (Figure 5c,d). However, LIMKi significantly enhanced TNFα-induced permeability in ECFCs (*p* < 0.05) (Figure 5c,d), reproducing the situation observed in ECFC Eng-siRNA (Figure 1c). Of note, TNFα stimulation led to a strong decrease in F-actin polymerization only in the absence of Eng or in LIMKi-treated ECFC, (Figure 5e–g), suggesting that Eng and cofilin are important players in actin rearrangement during TNFα stimulation. 

Our data suggest that the decrease of cofilin-phosphorylation observed in ECFC Eng-siRNA leads to an enhanced ECFC permeability under the inflammatory stimulus of TNFα. Since tubulin is another player in the barrier integrity, we analyzed tubulin post-translational modifications (PTM). Indeed, tubulin detyrosination is associated with longer-lived microtubules, whereas more dynamic microtubules are found to be mainly tyrosinated in contrast to acetylated microtubules that are less dynamic. Modification of these PTM contributes to endothelial barrier integrity. However, in the absence of Eng, no modification of PTM of the α-tubulin was observed. (Figure S5). Taken together, our data demonstrate that Eng regulates F-actin dynamic through cofilin regulation, which is in turn necessary to maintain ECFC permeability.

## 3. Discussion

For the past fifteen years, Eng has been described as an active angiogenic player. Recently, in human ECFCs, we described Eng as a regulator of vessel stabilization and interaction between EC and pericytes [11,30]. Our present study demonstrates that Eng is involved in ECFC cytoskeleton organization and permeability under inflammatory conditions involving the LIMK/cofilin pathway. Our findings are in line with the hypothesis of a second hit in HHT pathology [31]. Indeed, Eng might behave as an endothelial housekeeper against inflammation by regulating cell permeability.

TNFα is an inflammatory cytokine present in a soluble trimeric form of 55 KDa [32], which is released by the membrane-bound protease coined TACE (TNF-α-converting enzyme, also known as ADAM-17-A disintegrin and metalloproteinase 17) and capable of promoting a vascular hyperpermeability that induces gradual changes in several minutes—hours [33,34]. Interestingly, we find that in silico modeling predicts an interaction between TNFα and endoglin. To evaluate ECFC permeability in the present work, Eng-silenced and control ECFCs were stimulated with TNFα. The putative interaction between TNFα ligand and membrane Eng supports the hypothesis that TNFα would be less effective in the presence of Eng. Conversely, when Eng is completely or partially silenced, TNFα effect is fully active to increase cell permeability. Therefore, Eng could be considered as a membrane protective agent by limiting TNFα effects on endothelial permeability. Furthermore, the increased permeability of ECFC Eng-siRNA in the presence of TNFα was associated with a significant Ca^2+^ mobilization. The question that remains is why an altered permeability is observed only in the presence of TNFα when Eng is inhibited. It is known that Eng expression is upregulated in the EC of inflamed tissues with an associated inflammatory cell infiltrate [35] and also that there is a redistribution of Eng in cell–cell contact after TNFα treatment [10]. Independent experiments to investigate the interaction between endoglin and TNFα remain to be studied further. Vascular permeability has been associated with edema and endothelial dysfunction [36]. We recently described an increase in soluble Eng (sEng) in case of edema related to hemodynamic perturbation in cardiac assistance [36]. Since we described here Eng as a protective agent for vascular permeability maintenance, the increased level of sEng in plasma probably reflects Eng cleavage in EC. Regarding the new role of Eng described here, edema could be the consequence of this decreased endothelial expression of Eng [36].

ECFCs from HHT1 patients already showed disorganized and impaired tube formation [7,23]. The fragile cytoskeleton was attributed to an alteration of TGFβ pathways in HHT patients where a decreased Eng expression or impaired ALK1–ALK5 signaling were found, but other possible mechanisms were not considered. Recent evidence may shed new light on this question ,suggesting that Eng: (i) participates in canonical and non-canonical TGFβ signaling [9]; (ii) it may impact EC behavior via regulation of cell adhesion [10,11,30,37,38]; and (iii) it can influence ECFC adhesive properties [30,38]. Reorganization of the cytoskeleton and mainly actin dynamics is an important process regulating cell permeability, endothelial junctions, and cell deformation [24]. Since actin dynamics is regulated by the actin turnover-regulating protein cofilin [25], we hypothesized that the disorganized actin network observed in Eng-siRNA ECFC would depend on the activity of cofilin, which is modulated by Eng. A disorganized cytoskeleton is prone to cell breaking with changes in shear stress and may lead to vessel hemorrhages. ECFCs are a key mechanism in vascular remodeling and are involved in the adaptive process that normally occurs in response to long-term changes in hemodynamic conditions and in blood vessel repair. Due to the important changes observed in actin distribution when Eng is inhibited, we postulated a participation of Eng in the pathway of LIMK/cofilin. LIM kinase-1 and -2 are actin-binding kinases that phosphorylate members of the ADF/cofilin family of actin binding and filament-severing proteins. Previous studies have shown that the Eng cytoplasmic tail interacts with zyxin and the zyxin-related protein ZRP-1, which are both members of the LIM family of proteins involved in actin cytoskeleton organization [28,29]. Eng via its cytoplasmic domain interacts with LIM proteins, but the relation between cofilin and Eng remains incompletely clarified. The main cellular function of cofilin is to change cytoskeletal dynamics, in turn modulating cell motility and cytokinesis [24]. When not phosphorylated, cofilin stimulates the severing and depolymerization of actin and promotes actin turnover. In contrast, when phosphorylated at Ser3, cofilin enters an inactive state and loses its ability to bind actin. Consequently, actin filaments are stabilized and disorganized and accumulate in areas enriched in phosphorylated cofilin [25]. In ECFCs, the role of Eng on actin turnover was not proven nor investigated, and its correlation with permeability was not elucidated. We analyzed P-cofilin, a terminal effector of signaling cascades that evoke actin cytoskeletal rearrangement, showing that in ECFCs, Eng loss reduces P-cofilin concomitantly with tubulin and actin redistribution. These findings were further confirmed in mouse cell models (*Eng^+/−^* vs. *Eng^+/+^* MLEC). Our results suggest a dynamic actin turnover dysregulating ECFC cell–cell contact. Despite the fact that microtubules contribute to the dynamic reorganization of the endothelial cell cytoskeleton, we ruled out the possible Eng regulation on tubulin because no modifications of α-tubulin PTM were observed in the absence of Eng. 

In conclusion, our data demonstrate that Eng contributes to the ECFC cytoskeleton regulation and inflammation-induced permeability through a cofilin/actin signaling pathway. Indeed, when LIMK is inhibited, the ECFC phenotype and behavior is similar to that observed with ECFC Eng-siRNA. Accordingly, since inflammation could be a second hit in HHT-1, our results support the concept that Eng is a vascular housekeeper for permeability associated to inflammation in vascular disorders. A better understanding of the molecular mechanisms of Eng combined with preclinical models of inflammation and vasculopathy may help to identify new pharmacological approaches in inflammation-related pathologies.

## 4. Materials and Methods

### 4.1. ECFC Isolation, Culture, and Transfection

ECFCs were isolated from the adherent mononuclear cell (MNC) fraction as described [39]. Then, ECFCs were expanded on fibronectin (FN)-coated plates (1 μg/cm^2^; Millipore, Billerica, MA, USA) using EGM-2 medium (without hydrocortisone; Lonza, Walkersville, MD, USA) supplemented with 10% fetal bovine serum (FBS; Hyclone, Logan, UT, USA). ECFCs were used at passages P3–5 and at day <30. Endoglin-specific siRNA (Eng-siRNA; sc-35302, Santa Cruz Biotechnology, CA, USA) was used to silence human Eng. Briefly, 10 µM siRNA was mixed with the Dharmafect reagent (SO-2511539G Dharmacon, USA) to obtain transfection complexes, which were added to ECFCs in EGM2 medium in six-well plates. ECFCs transfected with scrambled siRNA (Scramble, Allstars Neg. control siRNA, Qiagen, Cambridge, MA, USA) were used as control (ctr-siRNA). To determine the efficiency of Eng suppression, immunofluorescence microscopy, flow cytometry, and Western blot analyses were used. 

### 4.2. MAEC and MLEC Isolation from Mouse Model of HHT1 (Eng^+/−^ Mice)

Mouse aortic endothelial cells (MAEC) and mouse lung endothelial cells (MLEC) from *Eng^+/+^* and *Eng^+/−^* mice were isolated and cultured as previously described [40].

### 4.3. Immunofluorescence Microscopy

MAECs, MLECs, or ECFCs were seeded (50,000 cells/mL per well) and cultured on chamber slides (Millicell EZ slide, Millipore). When confluent, cells were fixed with 4% paraformaldehyde (PFA) in PBS for 15 min at room temperature (RT), blocked with 1% BSA/PBS for 1 h, and permeabilized, when necessary, with 0.1% Triton X-100 in PBS for 5 min. For ECFC immunostaining, samples were incubated with mouse antibodies against human Eng (CD105-Alexa488, #MHCD10520 Invitrogen-Thermo Fischer Scientific, MA, USA; dilution 1:100), VE-cadherin (VE-CAD)/CD144 (Invitrogen, dilution 1:50) for 1 h at RT. In the case of VE-CAD, an additional incubation with secondary antibodies FITC-anti-mouse IgG (Vector, CA, USA, dilution 1:200) for 1 h at RT was carried out. For intracellular staining of actin and tubulin, cells were incubated with Alexa^®^ Fluor 546 or Alexa^®^ 488 conjugated to phalloidin (targeting actin; Invitrogen, dilution 1:20) for 20 min or with a mouse anti-α tubulin antibody (Sigma-Aldrich, MA, USA, T5168, dilution 1:100) for 1 h 30 min. The secondary goat anti-mouse Alexa^®^ Fluor 488 antibody (Invitrogen) was applied 1 h at RT. When indicated, cells were pretreated for 1 h with 20 ng/mL of TNFα (R&D, MN, USA). The LIMK inhibitor (LIMKi) (Millipore) was suspended in DMSO and preincubated with the cells at 10 μM for 10 min before TNFα treatment, DMSO alone being used as control. The Vectashield mounting medium for fluorescence with DAPI (H-1200, Vector) was used to counterstain nuclei. Alternatively, TO-PRO-3 (642/661, Invitrogen-Thermo Fischer Scientific, MA, USA) and IBDI mounting medium were used. Samples were analyzed by confocal microscopy (Confocal Laser Scanning Microscope, CLSM, Leica TCS SP5, Leica Biosystems France). To analyze co-localization of dual color fluorescence within microscopy images, the Pearson’s correlation coefficient was used by measuring the covariance between two signals as described [41]. Pearson’s coefficients were calculated using the plug-in JACoP from Image J, and correlation coefficients ranged from −1 (a perfect negative correlation) to +1 (a perfect positive correlation).

### 4.4. Immunofluorescence Flow Cytometry

Briefly, ECFCs in suspension (150,000–200,000 cells/mL) were first incubated with 1% BSA/PBS for 30 min at 4 °C and then with a mouse monoclonal antibody against human Eng (CD105-Alexa488, #MHCD10520 Invitrogen; dilution 1:50) for 1 h at 4 °C. After two washes with PBS at 4 °C, the mean fluorescence intensity (MFI) was measured with an Accuri flow cytometer (BD Biosciences, Le Pont de Claix, France). 

### 4.5. Angiogenesis Assays Using Cytodex

Three-dimensional fibrin gel assays were performed as previously described [42]. ECFCs were seeded (1 × 10^6^ cells per 2500 beads) onto Cytodex beads (Sigma-Aldrich) and embedded in a 2.5 mg/mL fibrin gel in the presence of EGM-2 medium in chamber slides (Millicell EZ slide, Millipore). Mesenchymal stem cells (40,000) were plated on the top of the gel as feeder cells. After 7 days in culture, feeders were trypsinized (10X trypsin, 5 min at 37 °C), and the fibrin gel was fixed with 4% PFA. Gels were stained with Alexa Fluor-488-conjugated phalloidin and TO-PRO-3 (642/661) (Invitrogen—Thermo Fisher Scientific, MA, USA). Images were acquired with a Leica Confocal laser scanning microscope TCS SP8. The number of sprouts and cumulative tube length per bead was measured using the Image J macro as described [34]. Images were taken using a confocal microscope (Leica SP-5) and video recording (Leica Las AF Lite). Two-hundred beads for different ECFC clones (*n* = 6) were evaluated.

### 4.6. ECFC Barrier Permeability Assay

The barrier function of human ECFC was evaluated using a real-time impedance-based cell analyzer (iCELLigence system, ACEA Biosciences), as previously described for a variety of endothelial cell types [43]. Briefly, 50,000 ECFCs were plated on each well of E-plates L8 (ACEA Biosciences) and cultured for 48 h in EGM-2 media (Lonza) and stimulated with 1, 10, 20, or 50 ng/mL of human recombinant TNFα (R&D). The concentration of 20 ng/mL was further chosen to perform all assays, as it was the minimum concentration that gave the more reproducible data for all the cell clones. The Cell Index (CI, a measure of cell impedance) was normalized at the time of TNFα challenge and was monitored every minute for 24 h. TNFα stimulation induced a drop in the normalized cell index (NCI) that was maximal at 12–16 h. Permeability was quantified by measuring the NCI at 16 h post-TNFα challenge.

### 4.7. Measurement of Intracellular Ca^2+^

For each assay, 50,000 ECFCs control-siRNA and eng-siRNA were plated on a 25 mm polysine-coated coverslip in a 6-well plate in 2 mL of EBM2 for 24 h. Then, cells were incubated at room temperature with the Ca^2+^-sensitive dye Oregon Green 488 BAPTA 1-AM (1 mM) for 45 min. Ca^2+^ mobilization from intracellular stores induced by TNF (20 ng/mL) was analyzed in Ca^2+^ free medium (100 µM EGTA) and in the presence of 300 µM extracellular Ca^2+^. Fluorescence was immediately recorded by an epifluorescence microscope (Nikon Eclipse TE2000-U) and a black and white CCD (CoolSNAP HQ Photometrics, Tucson, AZ, USA) camera at 2 s intervals using the Metamorph 7.0r1 software. Changes in Ca^2+^ signal intensity were calculated as the ratios of fluorescence of activated over non-activated cells, and the area below the curve for 2 min after agonist addition was chosen as an indicator of the Ca^2+^ response as previously reported [44].

### 4.8. Computational Analysis of Endoglin Structure

To build the structural model of Eng (UnitPROT #p17813), we used the PDB IDs 5HZW and 5HZV [4]. The ID #5HZW was used as reference structure for the alignment and for the orphan region 1 (OR1) and 2 (OR2) domains [4]. For the ID #5HZV, we considered the subunits of ZN-N (zona pelucida at the N-terminus) and ZP-C (zona pelucida at the C-terminus). To align and match the PDB structures, the UCSF Chimera visualization system [45] was used, and the missing part was obtained using homology models via MODELLER9 [46]. The homodimerization of Eng is mediated by C516–C516 and C582–C582 disulfide bonds [4,47]. Hence, we added these disulfide bonds to our model by imposing a distance of 2.05 Å. Finally, for the transmembrane part, we built a helical model based on this portion of the sequence. We combined the extracellular domain with the transmembrane one using USCF Chimera. In the absence of ligand, Eng seems to switch from an open to a closed form. Thus, for our simulations, we considered both the open and closed forms.

### 4.9. Coarse-Grain Molecular Dynamic Simulations for TNFα and Endoglin

We ran a simulation considering TNFα in its soluble form in interaction with Eng embedded in a single-component, homogenous dipalmitoyl-phosphatidyl-choline (DPPC) lipid membrane. As control, we also ran simulations of Eng alone and TNFα alone in the membrane. We considered two different starting configurations: one starting with Eng in open form and one starting with Eng in closed form. For the human TNFα (UniProt: P01375), six structures are available for the extracellular region (residues 57-233). Due the high sequence identity, we built a template model using the SWISS-MODEL [48] for residues 84–233. For the remaining residues of the extracellular part (residues 57–83), the Pep-FOLD software [49] was used. The MARTINI coarse-grained (CG) force field was used to model all the components of our systems: water, lipid membranes, proteins, and ions [50]. This choice allowed us to easily reach time scales of tens of microseconds. All the simulations were carried out with the Gromacs software package [51]. The conversion from all-atom to CG of the proteins was computed using the MARTINIZE tool [50]. Starting configurations for the membranes were generated with the INSANE tool [52]. The length of each simulation was 15 µs. CG simulations were performed in the NpT ensemble, with periodic boundary conditions in all dimensions. A leap-frog integrator with a time step of 30 fs was used. The temperature was set to 310 K using the Donadio–Bussi–Parrinello thermostat, with a time constant of 1 ps. The pressure was set to 1 bar using a Berendsen weak coupling algorithm in equilibration runs with a time constant of 12 ps and the Parrinello–Rahman [53] barostat for production runs with a time constant of 12 ps. Pressure control was always semi-isotropic, with z (the direction of the membrane normal) coupled independently of the X and Y axes. Some snapshots were converted from AA to CG using the BACKMAPPING tool [53], followed by 20,000 steps of minimization.

### 4.10. Immunoblotting

ECFCs (6-well plate, 500,000 cells/well) were lysed in SDS denaturing buffer (50 mM Tris, 100 mM NaCl, 50 mM NaF, 5 mM EDTA, 40 mM β-glycerophosphate, 100 μM phenylarsine oxide, 1% SDS, 5 μg/mL leupeptin, 10 μg/mL aprotinin, pH 7.4). Proteins were subjected to SDS-PAGE and transferred to nitrocellulose. The membranes were incubated with various primary antibodies to phospho-cofilin (Ser 3) (Cell Signaling, MA, USA #3311, rabbit antibody, 1/1000), cofilin (Cell Signaling, MA, USA #5175, monoclonal rabbit antibody, 1/1000), α-actin (R&D, UK #MAB8929, mouse monoclonal antibody, 1/40,000), α-tubulin (Abcam , Cambridge, UK #11304, mouse monoclonal antibody, 1/2000), acetylated α-tubulin (Sigma #T7451, mouse monoclonal antibody, 1/10,000), tyrosinated α-tubulin (Millipore #ABT171, rabbit, 1/2000), detyrosinated α-tubulin (Abcam #48389, rabbit, 1/1000) and Eng (mouse monoclonal antibody; P4A4) [11]. Secondary antibodies used were HRP-conjugated goat anti-mouse (Jackson Immunoresearch, PA, USA #115-035-003, 1/20,000) and HRP-conjugated goat anti-rabbit (Jackson Immunoresearch PA, USA #111-035-144, 1/20,000). Immunoreactive bands were visualized using Enhanced Chemiluminescence Detection Reagents (Pierce). Images of the chemiluminescent signal were captured using G:BOX Chemi XT16 Image Systems and quantified using ImageJ 1.53 software.

### 4.11. Statistical Analysis

Data are shown as mean ± SEM. Significant differences were identified by ANOVA followed by Fisher’s protected least-significant difference test. Intergroup comparisons were based on the Mann–Whitney non-parametric test. For permeability assays and Western blot analysis, Student’s t-test was used. Wilcoxon’s test was applied to assays measuring intracellular Ca^2+^. All statistical tests were performed using the Stat View 5.0 software package (SAS, Cary, NC, USA). Differences with *p* < 0.05 were considered as statistically significant. * *p* < 0.05, ** *p* < 0.01, *** *p* < 0.001.

## Data Availability

All data are available upon request.

## Supplementary Materials

The following are available online at https://www.mdpi.com/article/10.3390/ijms22168837/s1, Figure S1: Endoglin silencing with siRNA, Figure S2: Effect of endoglin silencing on calcium ion mobilization, Figure S3: Predicted contact maps of endoglin and TNFα, Figure S4: Three-dimensional (3D) models of TNFα ligand and membrane endoglin, Figure S5: Endoglin is not involved in tubulin post-translational modifications of ECFC. Video S1: Cytodex on siEng ECFC, Video S2: Cytodex on siSCR ECFC, Video S3: Calcium evaluation on siSCR ECFC, Video S4: Calcium evaluation on siEng ECFC, Video S5: TNFα–endoglin open map, Video S6: TNFα–endoglin open contacts, Video S7: TNFα–endoglin closed map, Video S8: TNFα–endoglin closed contacts, Video S9: Endoglin alone MAP, Video S10: TNFα alone MAP.

## Abbreviations

ctr-siRNA	scrambled siRNA used as control
EC	Endothelial cells
ECFC	Endothelial colony forming cells
Eng	Endoglin
Eng-siRNA	Endoglin-specific siRNA
HHT	Hereditary Hemorrhagic Telangiectasia
LIMKi	LIM Kinase inhibitors
MAEC	Mouse aortic endothelial cells
MLEC	Mouse lung endothelial cells
PTM	Post-translational modifications
TGF-β	Transforming growth factor-β
TNFα	Tumor necrosis factor-α
