# Supplementary material for: Endoglin Is an Endothelial Housekeeper against Inflammation: Insight in ECFC-Related Permeability through LIMK/Cofilin Pathway
Source: Int J Mol Sci. 2021 Aug 17;22(16):8837. doi: 10.3390/ijms22168837 (PMC8396367; doi:10.3390/ijms22168837)
Supplement: Supplementary file 1 [file ijms-22-08837-s001.zip › Supp Mat REV/Supplementary Materials IJMS v2.pdf]

International Journal of *Molecular Sciences*

MDPI

Supplementary Materials

Endoglin is an endothelial housekeeper against inflammation: insight in ECFC related permeability through LIMK/cofilin pathway

Elisa Rossi [1,2,*], Alexandre Kauskot [3], François Saller [3], Elisa Frezza [1,4], Sonia Poirault-Chassac [1,2], Anna Lokajczyk [1,2], Pierre Bourdoncle [5], Bruno Saubaméa [1,6], Pascale Gaussem [1,2,7], Miguel Pericacho [8], Regis Bobe [3], Christilla Bachelot-Loza [1,2], Carmelo Bernabeu [9], Samuela Pasquali [1,4], and David M. Smadja [1,2,7,10]

1 University of Paris, Faculty of Pharmacy, F-75006 Paris, France; da-vid.smadja@aphp.fr (D.M.S.); chris-til-la.bachelot-loza@parisdescartes.fr (C.B.-L.); pascale.gaussem@aphp.fr (P.G.); sonia.poirault-chassac@inserm.fr (S.P.-C.)

2 IThEM, Inserm UMR_S1140, Paris, France

3 HITh, UMR_S 1176, INSERM, University Paris-Saclay, F-94270 Le Krem-lin-Bicêtre, France; alexandre.kauskot@inserm.fr (A.K.)

4 Cible Thérapeutique et Conception de Médicaments UMR 8038 CNRS, Paris, France

5 Plate-forme IMAG'IC Institut Cochin Inserm U1016-CNRS UMR8104, Uni-versité Paris Descartes, Paris, France

6 UMR-S 1144, Paris, France

7 AP-HP, Hôpital Européen Georges Pompidou, Hematology Department, 75015 Paris, France

8 Department of Physiology and Pharmacology, Universidad de Salamanca, Salamanca, Spain; pericacho@usal.es (M.P.)

9 Centro de Investigaciones Biológicas Margarita Salas, Consejo Superior de Investigaciones Científicas (CSIC) and Centro de Investigación Biomédica en Red de Enfermedades Raras (CIBERER), Madrid 28040, Spain; bernabeu.c@cib.csic.es (C.B.)

10 Biosurgical Research lab (Carpentier Foundation), Paris, France

* Correspondence: elisa.rossi@parisdescartes.fr;

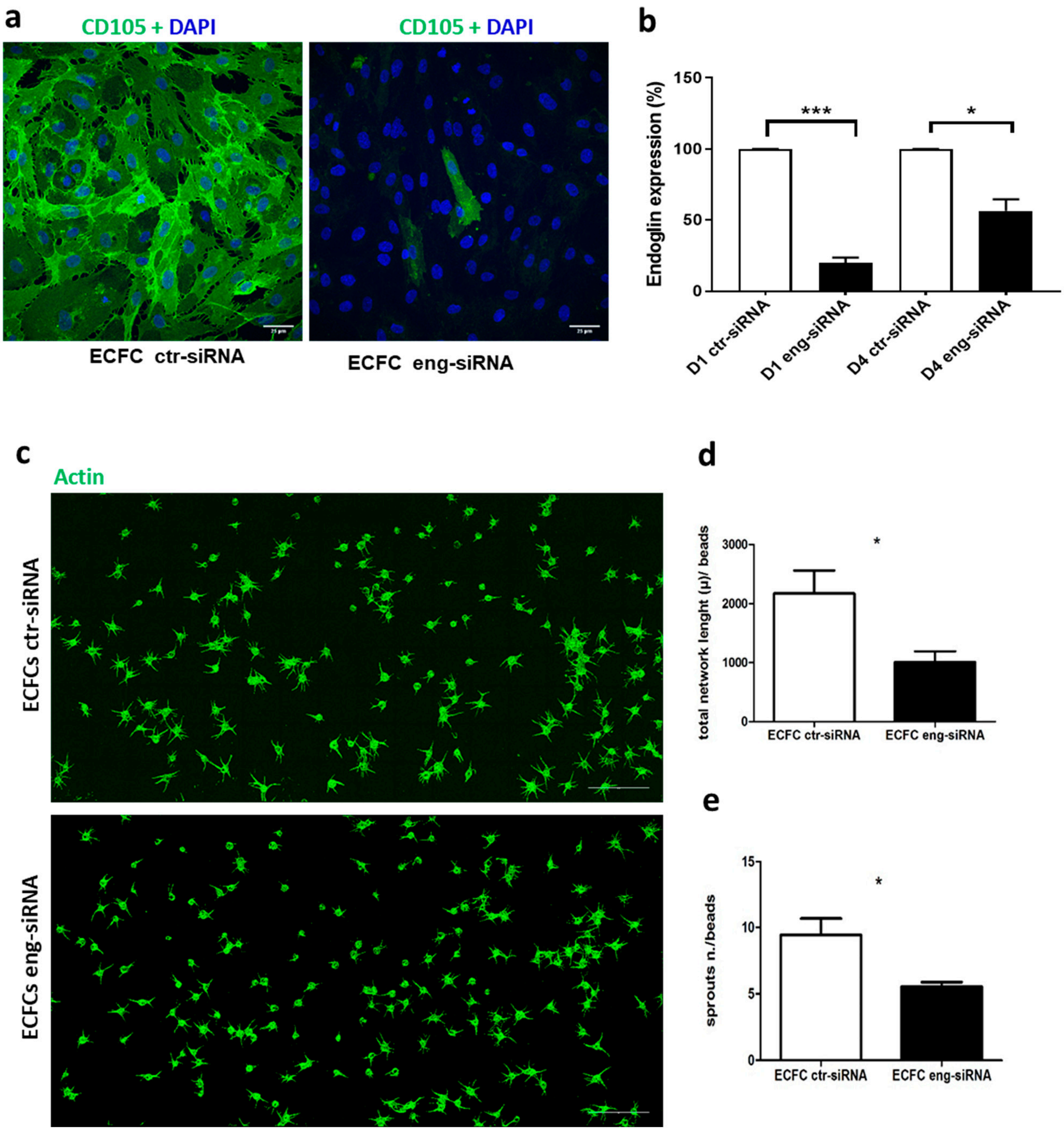

Figure S1. Endoglin silencing in ECFCs. Eng inhibition in ECFCs was carried with siRNA and endoglin expression was analyzed by immunofluorescence microscopy (**a**) and quantified by immunofluorescence flow cytometry, (**b**) showing the efficacy of siRNA silencing at day 1 (D1) and day 4 (D4). (**c**) Macro of the beads quantified representing ECFC control and ECFC eng-siRNA. In green, F-actin (Alexa 488). (**d**) Quantification shows a significant reduction in sprout length (*p<0.05) of ECFC eng si-RNA. (**e**) Quantification of sprouts/beads shows less sprouting in ECFC eng-siRNA than controls (*p<0.05).

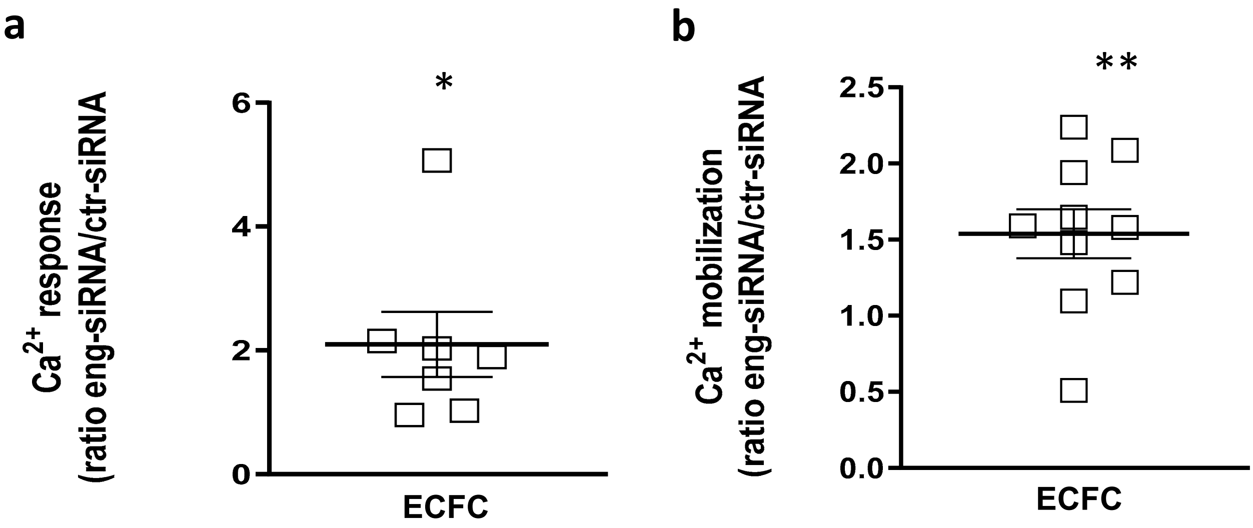

Figure S2. Effect of endoglin silencing on calcium ion mobilization. ECFC-ctr and ECFC eng-siRNA were loaded with the cytosolic Ca^{2+} fluorescent probe Oregon-Green-488 BAPTA-AM and Ca^{2+} mobilization of intracellular Ca^{2+} was assessed after stimulation with 20 ng/mL TNFα (**a**) in the presence of external Ca^{2+} (100 µM EGTA) or (**b**) in the absence of external Ca^{2+}. Both ratios of Ca^{2+} responses of Eng-siRNA over ECFC-ctr (either global Ca^{2+} responses, in the presence of external Ca^{2+} or Ca^{2+} mobilization, in the absence of external Ca^{2+}) are displayed as dot plots (n=7 and n=10, respectively).*$p<0.05$, **$p<0.01$ (paired t-test).

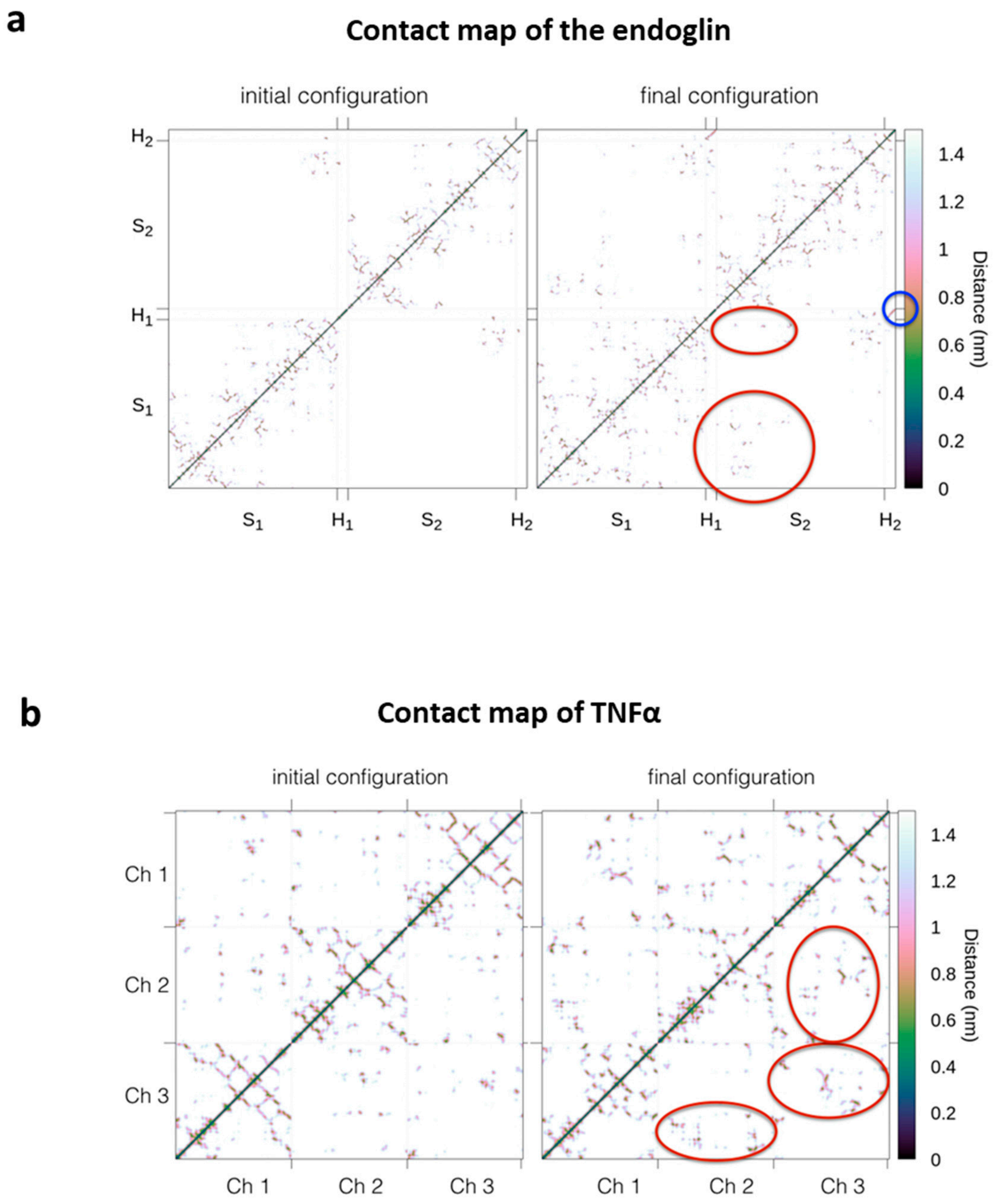

Figure S3. Predicted contact maps of endoglin and TNFα. (**a**) Contact map of the endoglin simulation. In the initial configuration (left) the two arms are separated from each other, while during the simulation (left) we can observe contacts both of the helices embedded in the membrane (H1 and H2, blue circle) and between the soluble parts outside the membrane (S1 and S2, red circles). (**b**) TNFα ligand. We observe the three chains of TNFα ligand, Ch1, Ch2 and Ch3, structured internally as β-sheets that all interact with each other as indicated by the red circles. During the course of the simulation some of the internal structuring of each chain is lost in favor of an increased interaction with other chains.

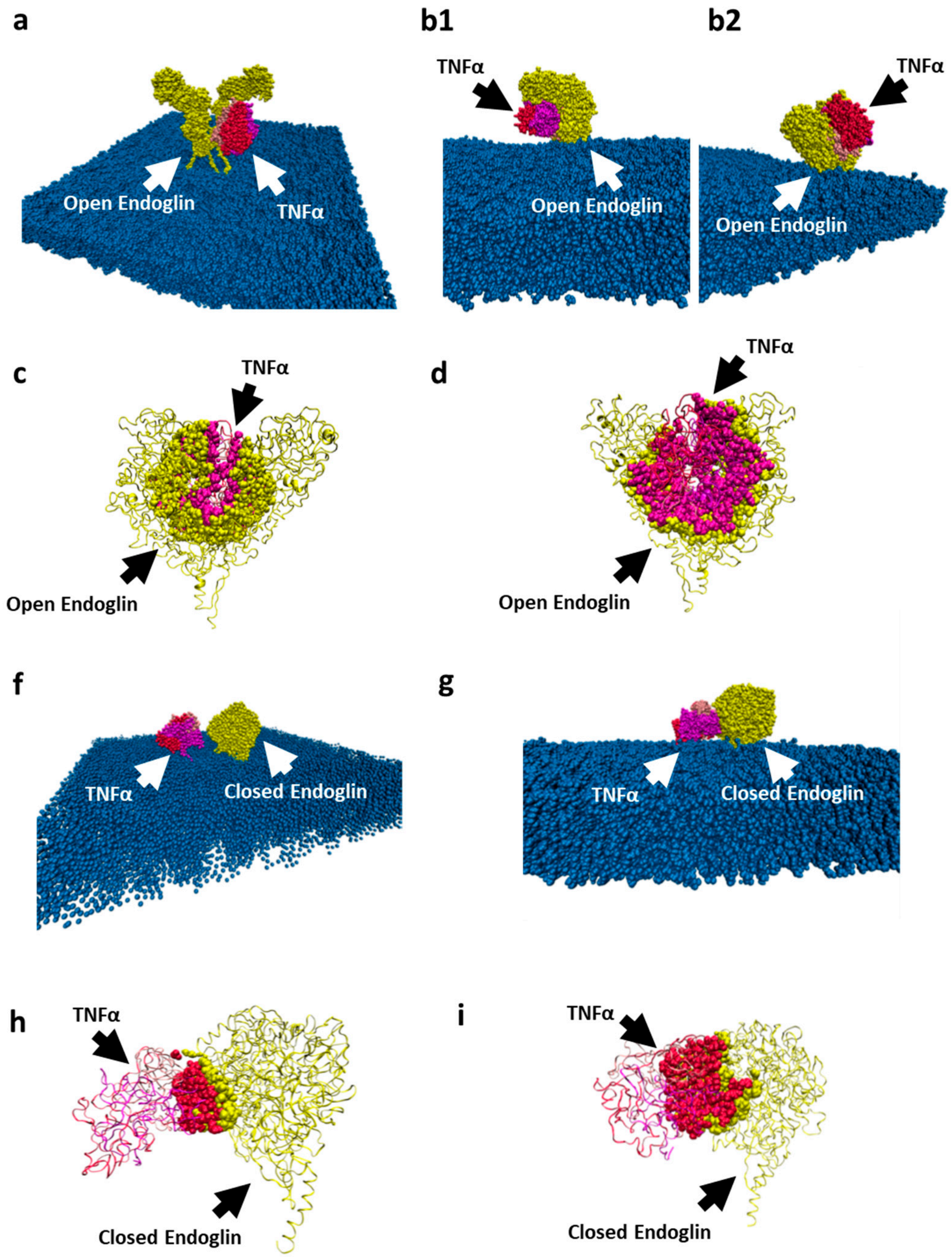

Figure S4. 3D models of TNFα ligand and membrane endoglin. TNFα ligand is represented in pink and membrane endoglin in yellow. (**a**) At the initial configuration the two molecules are separated and the endoglin is in an open configuration. (**b1** and **b2**) two angles of the late configuration to show that TNFα inserts deeply into the endoglin cavity. (**c**,**d**) Front and back view of the TNFα-endoglin complex. Surface contact highlights 137 contacts. (**f**) Initial configuration. The two molecules are separated and endoglin is in closed (folded) configuration. (**g**) In the late configuration the two molecules aggregate permanently. (**h**) In the early simulation, 28 TNFα residues are in contact with endoglin. (**i**) In the late simulation 54 TNFα residues are in contact with endoglin.

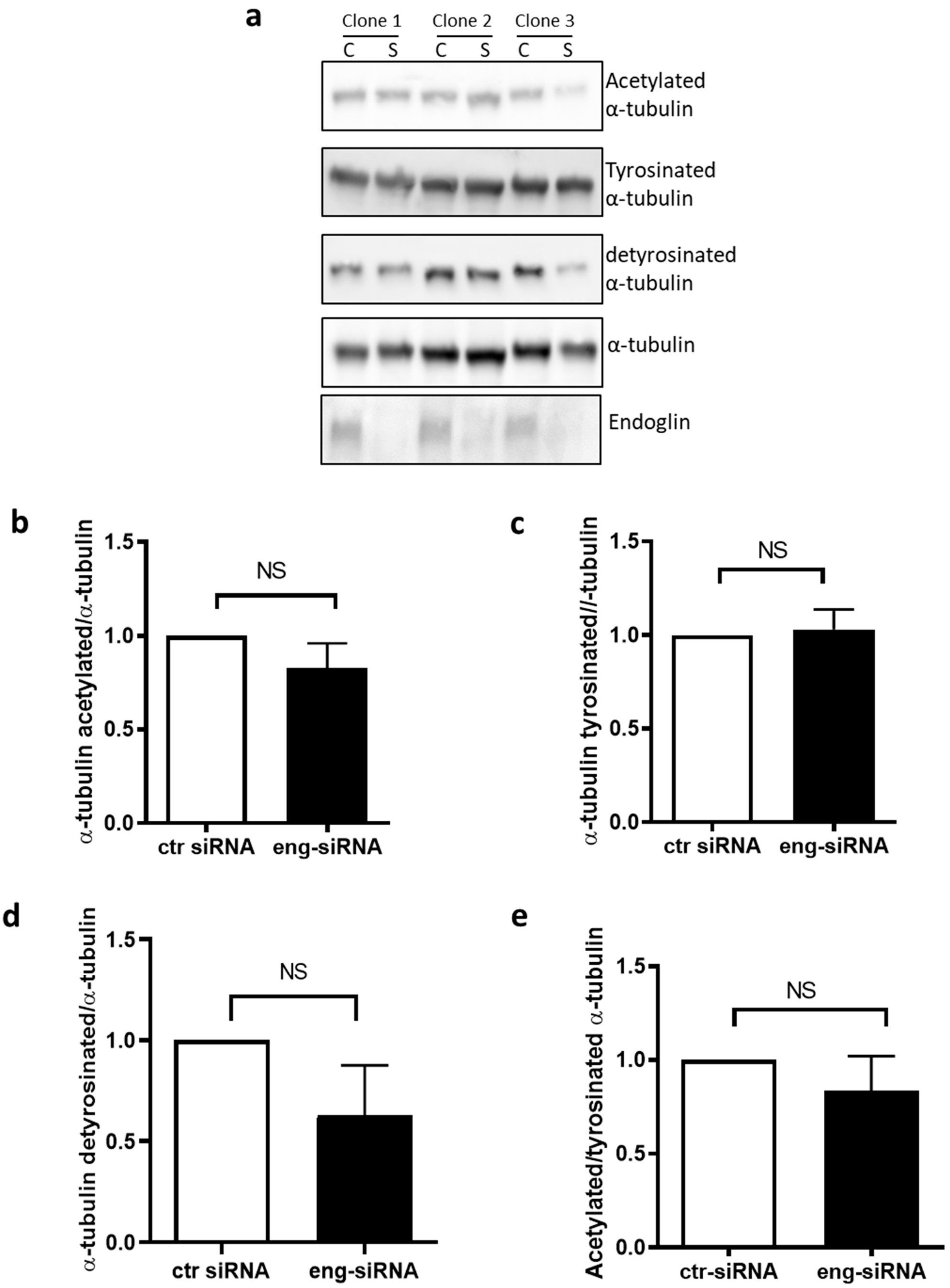

Figure S5. Endoglin is not involved in in tubulin post-translational modifications of ECFC. (**a**) Three different clones of ECFC were transfected with ctr-siRNA (C) or Eng-siRNA (S) and total extracts subjected to Western blot analysis using antibodies to acetylated, tyrosinated, detyrosinated or total α-tubulin, and to endoglin, as indicated. (**b-e**) Quantification of the different ratios of acetylated, tyrosinated, detyrosinated and total α-tubulin, as indicated. No significant differences were found between all conditions analyzed.
